# Do alternative methods for analysing count data produce similar estimates? Implications for meta-analyses

**DOI:** 10.1186/s13643-015-0144-x

**Published:** 2015-11-17

**Authors:** Peter Herbison, M. Clare Robertson, Joanne E. McKenzie

**Affiliations:** Department of Preventive and Social Medicine, Dunedin School of Medicine, University of Otago, PO Box 913, Dunedin, 9054 New Zealand; Department of Medicine, Dunedin School of Medicine, University of Otago, PO Box 913, Dunedin, 9054 New Zealand; School of Public Health and Preventive Medicine, Monash University, The Alfred Centre, 99 Commercial Road, Melbourne, Victoria 3004 Australia

**Keywords:** Count outcomes, Meta-analysis, Methods of analysis, Rates

## Abstract

**Background:**

Many randomised trials have count outcomes, such as the number of falls or the number of asthma exacerbations. These outcomes have been treated as counts, continuous outcomes or dichotomised and analysed using a variety of analytical methods. This study examines whether different methods of analysis yield estimates of intervention effect that are similar enough to be reasonably pooled in a meta-analysis.

**Methods:**

Data were simulated for 10,000 randomised trials under three different amounts of overdispersion, four different event rates and two effect sizes. Each simulated trial was analysed using nine different methods of analysis: rate ratio, Poisson regression, negative binomial regression, risk ratio from dichotomised data, survival to the first event, two methods of adjusting for multiple survival times, ratio of means and ratio of medians. Individual patient data was gathered from eight fall prevention trials, and similar analyses were undertaken.

**Results:**

All methods produced similar effect sizes when there was no difference between treatments. Results were similar when there was a moderate difference with two exceptions when the event became more common: (1) risk ratios computed from dichotomised count outcomes and hazard ratios from survival analysis of the time to the first event yielded intervention effects that differed from rate ratios estimated from the negative binomial model (reference model) and (2) the precision of the estimates differed depending on the method used, which may affect both the pooled intervention effect and the observed heterogeneity.

The results of the case study of individual data from eight trials evaluating exercise programmes to prevent falls in older people supported the simulation study findings.

**Conclusions:**

Information about the differences in treatments is lost when event rates increase and the outcome is dichotomised or time to the first event is analysed otherwise similar results are obtained. Further research is needed to examine the effect of differing variances from the different methods on the confidence intervals of pooled estimates.

**Electronic supplementary material:**

The online version of this article (doi:10.1186/s13643-015-0144-x) contains supplementary material, which is available to authorized users.

## Background

Often the outcomes measured in medical research are count outcomes. Typically, these measure the number of times a particular event happens to an individual in a defined period. Examples of count outcomes include the number of falls by the individual, the number of asthma exacerbations or the number of incontinence episodes. These outcomes are commonly measured in randomised controlled trials (RCTs) to determine the effect of an intervention.

There are many ways of summarising the difference between interventions when the outcome is a count outcome [1–3], such as:

- A simple rate ratio—the ratio of the number of events per person time at risk in each of the treatment groups.
- A rate ratio calculated from the Poisson regression family—such as Poisson and negative binomial.
- A risk ratio after the data are dichotomised into those with and without the event.
- A hazard ratio using the time to the event—either the time to the first event or using a method that copes with multiple times to events.
- A difference in means that treats the data as continuous and is compared using a *t* test or linear regression. More recently, the ratio of means has been used [4]. These analyses cause few problems for count outcomes with a high mean, such as pulse rate, as the Poisson distribution with a high mean approximates a normal distribution. In practice, however, this approach is often used on data with lower means.
- A difference in medians tested by a non-parametric test such as the Wilcoxon rank sum test or the ratio of medians.

The variety of analytic methods used in RCTs with count outcomes causes difficulties when carrying out a meta-analysis. In addition to the usual problems of heterogeneity arising from populations and treatments, there is heterogeneity in outcomes and analysis methods used across RCTs to evaluate the effect of the intervention. This raises a key question of whether the results from these alternative methods of analysis are comparable enough (exchangeable) to be combined in a meta-analysis.

This paper describes a simulation study designed to see whether mixing the results of different methods of analysis could give reasonable answers in a meta-analysis.

Falling is a major health problem for older people, with approximately 30 % of people over the age of 65 falling each year, with many falls resulting in injury and hospitalisation. The 2009 Cochrane systematic review “Interventions for preventing falls in older people living in the community” included 43 trials that assessed the effect of exercise programmes [5]. The two primary outcomes in this review were the rate of falls and the proportion of fallers. Twenty-six of the 43 studies contributed to the rate of falls meta-analysis, and 31 to the number of fallers. Some studies could not be used because of the way the data were analysed and presented. We asked for individual patient data from randomised trials included in this systematic review, analysed them in different ways and compared the resulting meta-analyses.

## Methods

### The simulation study

Data sets for a two-group parallel RCT with varying parameters were created. The size of each group was randomly chosen from a normal distribution with a mean of 100 and a standard deviation of 2. This kept the sample sizes of the two arms approximately equal and was large enough to provide stable estimates of the difference between the groups. The number of events experienced for each individual was randomly selected from a Poisson distribution with various means covering the range of potential rates that might be expected in practice. There were two groups of simulations, one with a moderate effect of the intervention, with a rate reduction of approximately 30 % in each of the chosen base values, and one with only a small random difference between groups. There were four groups of simulations ranging from a mean of 0.15 to a mean of 7 for the Poisson distributions when there was no overdispersion. As it is common for count data to have some overdispersion, this was built into the data sets by including 0, 20 and 40 % of the numbers of events drawn from Poisson distributions with a higher mean (representing no, moderate and high overdispersion, respectively). Overdispersion was built into both arms of the trial so that the relative differences between the arms would remain approximately the same. As all methods were used on each data set, these minor differences are not problematic. The combinations of parameters for the simulation scenarios with the approximately 30 % reduction in event rate are shown in Table 1. The simulations with no reduction used the rates, with minor random perturbations, in the control group for both arms.

**Table 1:** Means of the poisson distributions used in the simulations

	Base values		Overdispersed values (0 %—no overdispersion, 20 %—moderate overdispersion and 40 %—high overdispersion from these Median, 2.5 and 97.5 centilesdistributions)	
	Control	Treatment	Control	Treatment
Very low	0.2	0.15	0.6	0.4
Low	0.5	0.35	0.8	0.6
Medium	2	1.5	3	2.5
High	7	5	10	7

The treatment period was set at 365 days. About 20 % of observations were randomly chosen to be lost to follow-up after a randomly chosen number of days. The number of days to the events experienced was chosen from a uniform distribution. The simulation started by choosing the sample size and then the number of events in each arm and the follow-up period. Then the time to each of the events was generated. This was followed by the different analyses, and the results were stored on a file. There were 10,000 replications of each simulation scenario. Stata code for one of the simulations is available in the Additional file 1.

Each data set was analysed in nine ways to obtain the following estimates of the intervention effect:

1. Simple rate ratio (RaR) calculated from count data (the ratio of the number of events divided by person time of follow-up in the intervention arm to the control arm)
2. Rate ratio estimated from Poisson regression [2, 6]
3. Rate ratio estimated from the negative binomial regression [7]
4. Risk ratio (RR) calculated from dichotomised data
5. Hazard ratio (HR) estimated from survival analysis for time to the first event [8]
6. Hazard ratio for multiple events estimated using the marginal model [9]
7. Hazard ratio for multiple events estimated using the Andersen-Gill model [9, 10]
8. The ratio of the mean number of events [4]
9. The ratio of the median number of events.

The negative binomial regressions used the mean-dispersion model. The marginal model for repeated time to events assumes that individuals are at risk for every event from the time of study entry, whereas the Andersen-Gill method assumes that people are at risk for the second event only from the time they had the first event. People with *n* events have *n* + 1 records in the file, *n* ending in the event and the *n* + 1th censored at the end of the follow-up. Adjustment is made for multiple records per person in both of these models. See Robertson et al. [11] for more details.

Where possible, information about the 20 % of observations lost to follow-up was included in the analysis [12]. Simple rate ratios were calculated using person days of follow-up, and the Poisson and negative binomial regression models allowed for varying lengths of follow-up through inclusion of an offset in the model. The survival models allow for varying lengths of follow-up through censoring. However, it is not possible to allow for follow-up time for intervention effect estimates 4, 8 and 9.

Ratios of means and medians (estimates 8 and 9) were used in preference to differences in means and medians, since these ratio measures were comparable with the other estimates of intervention effect although they would not usually be used in practice.

The results of the simulations were examined in several ways. Histograms of the results were produced, with the median, 2.5th and 97.5th centiles of these distributions plotted using forest plots. In addition, to examine the exchangeability of the methods, we compared the estimated intervention effect from each method of analysis with the estimated rate ratio from the negative binomial regression using two metrics. The first metric compared the mean of the differences in the two estimates (for ease of interpretation) across the 10,000 replicates, while the second metric compared the mean of the ratios of the two estimates (since the underlying scale is relative). The rate ratio from the negative binomial regression model was chosen as the reference estimate. The negative binomial model is a more general model compared with the Poisson regression model that relaxes the strong assumption that the underlying rate of the outcome is the same for each included participant. The negative binomial model requires the additional estimation of a dispersion parameter (which will make it less efficient than the Poisson model in the absence of overdispersion); however, the model is theoretically more plausible [11, 13, 14]. The mean and standard deviation of the differences in the estimates are presented in the “Results” section and of the ratios in the Additional file 1. The interpretation of a positive mean difference is that the estimate for the comparison method was closer to 1 compared with the estimate from the negative binomial regression. Mean differences enable a judgement about whether there is, on average, an important difference between the estimates calculated from each of the analytical methods and the comparison method. The standard deviation of the differences gives an indication of how close the average result is to the negative binomial rate ratio, with large values indicating that the estimates are not always close and that for any particular trial, the use of an alternative method may result in an importantly different estimate of intervention effect.

Stata V13 (StataCorp, College Station, TX, USA) was used for all simulations.

### The case study

All of the corresponding authors of the trials that contributed to the comparison of multiple component group or home-based exercise programmes versus no exercise programme in the 2009 Cochrane systematic review were invited by e-mail to contribute to this part of the study.

The e-mail provided authors with information and preliminary results of the simulation study and informed them of the purpose of this empirical study. Authors were then asked whether they would consider taking part in the empirical study. Those opting to participate in the empirical study were sent a second e-mail and given the option of undertaking a series of analyses themselves (and contributing the results) or providing de-identified individual participant data sets, which we would analyse. All authors who agreed to take part chose the latter option.

Each data set was analysed to estimate the effect of exercise versus no exercise using a (1) simple RaR, (2) RR calculated from the dichotomised outcome (fallers and non-fallers), (3) RaR estimated from Poisson regression, (4) RaR estimated from the negative binomial regression and (5) the ratio of means. The median number of falls in all groups was zero, so the ratio of medians could not be computed. Nor was it possible to undertake survival analyses because most studies either did not collect the times of the falls or did not provide this data. One trial was cluster randomised and so the Poisson regression and negative binomial regression were allowed for the potential within-cluster correlation [15].

For each analytical method, estimates of the intervention effect were pooled using both fixed and random effects meta-analytic models using the metan routine in Stata [16]. Fixed effect meta-analyses used the method of Mantel and Haenszel [17], and the random effects models used the method of DerSimonian and Laird [18].

## Results

The following are results for the simulations with the approximately 30 % reduction in treatment effect.

### Simulations with a very low mean

**Fig. 1:**
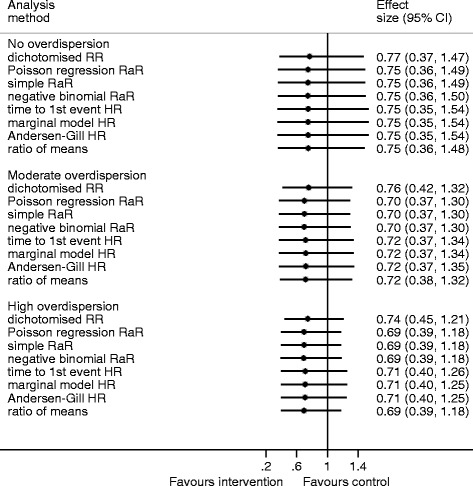
Median, 2.5 and 97.5 centiles (percentage-based confidence intervals) from the simulation results for the very low mean (control 0.2, treatment 0.15)

Simulations with a very small mean and no overdispersion yielded estimates for all analytical methods that were similar to the negative binomial rate ratio (Fig. 1, Table 2). The percentile-based confidence intervals (CIs) around the estimates are very similar for all the methods (Fig. 1). The full distributions of the results are given in Additional file 1: Figures 1a to 1d. Dichotomising the data into event/no event yielded the largest difference, with an average RR of 0.8088 compared with 0.7941 from the negative binomial regression (an increase of 3 % (Additional file 1: Table S1)), but this is unlikely to change the interpretation of the result. The 95 % CIs become narrower as overdispersion increased but this was less so for the three survival analysis methods (Fig. 1). While the estimates from the other methods seem to be little affected by overdispersion, dichotomising the outcome yields a RR closer to 1 by 0.0481 for high overdispersion (an increase of 8 %), which may impact on clinical interpretation. The HR for time to the first event is the second most different result (0.7998 vs negative binomial 0.7941, 1 % increase), and this increases slightly with overdispersion (to a 4 % increase). Unsurprisingly, the estimates from the Poisson regression and the simple RaR are always exactly the same. Dichotomising the data, and the three survival analysis methods, have the largest standard deviations, suggesting that for any particular data set, the estimates computed by these analytical methods may differ substantially from the negative binomial RaR. The ratio of medians is impossible to calculate for low means as the median is zero if fewer than 50 % of participants in either group do not have the event.

**Table 2:** Simulation results for the very low mean (control 0.2, treatment 0.15)

	No overdispersion		Moderate overdispersion		High overdispersion	
	RaR^a^ = 0.79		RaR^a^ = 0.73		RaR^a^ = 0.72	
	Mean difference^b^	SD	Mean difference	SD	Mean difference	SD
Dichotomise RR	0.01	0.09	0.05	0.09	0.05	0.09
Poisson RaR	<0.01	0.01	<0.01	0.01	<0.01	0.01
Simple RaR	<0.01	0.01	<0.01	0.01	<0.01	0.01
Time to the first event HR	0.01	0.09	0.02	0.10	0.02	0.09
Marginal model HR	0.01	0.09	0.02	0.09	0.02	0.09
Andersen-Gill HR	<0.01	0.09	0.02	0.09	0.02	0.09
Ratio of means	<0.01	0.03	0.02	0.03	<0.01	0.03
Ratio of medians	Not possible as both medians are zero					

*SD* standard deviation

^a^Rate ratio from negative binomial regression model

^b^Mean of the estimate minus negative binomial rate ratio

### Simulation scenarios with a low mean

**Fig. 2:**
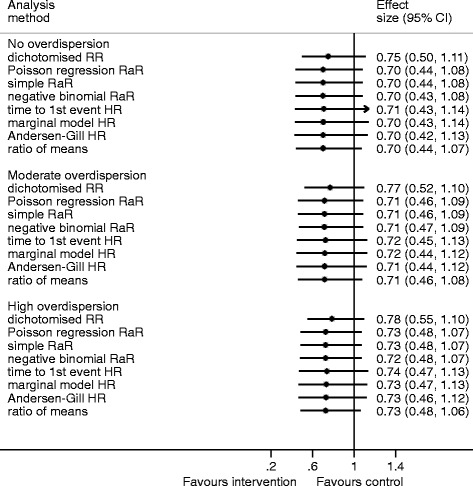
Median, 2.5 and 97.5 centiles (percentage-based confidence intervals) from the simulation results for the low mean (control 0.5, treatment 0.35)

With a slightly larger mean, the intervention effect estimates based on the dichotomised outcomes were, on average, importantly closer to the null compared with those estimated from the negative binomial regression model (Fig. 2, Table 3). The width of the percentile-based CIs of the estimated intervention effects were similar across the methods, although the width for the dichotomised outcome was slightly narrower (Fig. 1). The dichotomised RR was 8–9 % larger than the negative binomial RaR (Additional file 1: Table S2). Results for the other methods are similar to those using the lower mean. The histograms for these results are in Additional file 1: Figures 2a–d.

**Table 3:** Simulation results for the low mean (control 0.5, treatment 0.35)

	No overdispersion		Moderate overdispersion		High overdispersion	
	RaR^a^ = 0.71		RaR^a^ = 0.73		RaR^a^ = 0.74	
	Mean difference^b^	SD	Mean difference	SD	Mean difference	SD
Dichotomise RR	0.05	0.08	0.05	0.08	0.06	0.08
Poisson RaR	<0.01	0.06	<0.01	0.01	<0.01	0.01
Simple RaR	<0.01	0.06	<0.01	0.01	<0.01	0.01
Time to the first event HR	0.01	0.08	0.01	0.09	0.02	0.09
Marginal model HR	0.01	0.08	0.01	0.08	0.01	0.08
Andersen-Gill HR	0.01	0.08	0.01	0.08	0.01	0.08
Ratio of means	<0.01	0.03	<0.01	0.03	<0.01	0.03
Ratio of medians	Only possible for 292, 717 and 2087 of the 10,000 simulations, respectively					

*SD* standard deviation

^a^Rate ratio from negative binomial regression model

^b^Mean of the estimate minus negative binomial rate ratio

### Simulation scenarios with a moderate mean

**Fig. 3:**
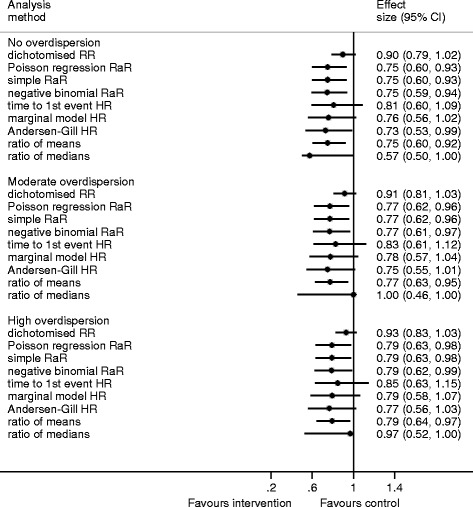
Median, 2.5 and 97.5 centiles (percentage-based confidence intervals) from the simulation results for the moderate mean (control 2, treatment 1.5)

When the mean in the control group is 2, dichotomising the data produces an estimate of effect much closer to 1 than the negative binomial RaR (Fig. 3, Table 4). The percentile-based confidence interval around the dichotomised RR is narrower than the other CIs (Fig. 3). The HR estimates from time to the first event analyses also move closer to 1, and the differences between the HR estimates and the RaR are more variable. Adjusting for multiple survival times, by using the marginal or Andersen-Gill method, seems to lessen the difference, but the differences have relatively large standard deviations, so individual intervention effects may be quite discrepant. The Andersen-Gill method yields estimates that are slightly further from 1 than the negative binomial regression estimates, but usually not enough to influence interpretation. The ratio of medians produces an average estimate closer to 1 than the negative binomial RR. The distribution of the ratio of medians is highly concentrated at discrete values (Additional file 1: Figure S3a, b, and c). The differences have the largest standard deviation across all levels of overdispersion, so that results from individual studies using this method may differ importantly from the negative binomial estimate.

**Table 4:** Simulation results for the moderate mean (control 2, treatment 1.5)

	No over dispersion		Moderate overdispersion		High overdispersion	
	RaR^a^ = 0.75		RaR^a^ = 0.77		RaR^a^ = 0.79	
	Mean difference^b^	SD	Mean difference	SD	Mean difference	SD
Dichotomise RR	0.15	0.07	0.14	0.08	0.14	0.08
Poisson RaR	<0.01	0.01	<0.01	0.01	<0.01	0.01
Simple RaR	<0.01	0.01	<0.01	0.01	<0.01	0.01
Time to the first event HR	0.07	0.11	0.06	0.11	0.07	0.12
Marginal model HR	0.02	0.09	0.01	0.09	0.01	0.10
Andersen-Gill HR	−0.01	0.08	−0.02	0.09	−0.02	0.09
Ratio of means	<0.01	0.03	<0.01	0.04	<0.01	0.04
Ratio of medians	0.05	0.19	0.09	0.17	0.07	0.14

*SD* standard deviation

^a^Rate ratio from negative binomial regression model

^b^Mean of the estimate minus negative binomial rate ratio

### Simulation scenarios with a large mean

**Fig. 4:**
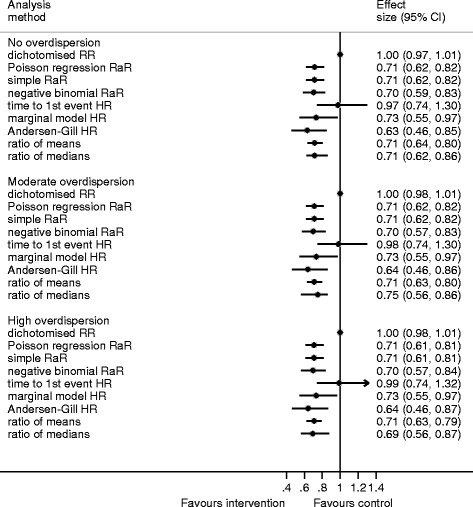
Median, 2.5 and 97.5 centiles (percentage-based confidence intervals) from the simulation results for the high mean (control 7, treatment 5)

With a mean in the control of 7, dichotomising these data sets yielded, on average, RRs close to 1 (Fig. 4, Table 5). The percentile-based CI is very narrow (Fig. 4). The time to the first event analysis was also suggestive of no intervention effect. The confidence intervals around the negative binomial RaR are wider than those around the simple RaR and the Poisson RaR. The percentile-based confidence intervals for the three survival analyses were wider than the other confidence intervals (Fig. 4). Adjusting for multiple survival times gave estimates closer to the negative binomial RaR, with the marginal model closer than Andersen-Gill. The results for the Andersen-Gill method are even further from one than the negative binomial RaR than with a smaller mean, yielding estimates that are importantly different between the two analytical methods. The standard deviations for the differences between the HR and the estimate from the negative binomial model in both of the marginal and Andersen-Gill methods were large. The ratio of medians yields similar estimates, on average, compared with the negative binomial RaR in scenarios with large means with confidence limits similar to those from the negative binomial regression (Fig. 4). This is despite the distribution of the ratios of medians being very non-normal.

**Table 5:** Simulation results for the high mean (control 7, treatment 5)

	No overdispersion		Moderate overdispersion		High overdispersion	
	RaR^a^ = 0.71		RaR^a^ = 0.70		RaR^a^ = 0.70	
	Mean difference^b^	SD	Mean difference	SD	Mean difference	SD
Dichotomise RR	0.29	0.06	0.30	0.07	0.30	0.07
Poisson RaR	0.01	0.02	0.01	0.03	0.01	0.03
Simple RaR	0.01	0.02	0.01	0.03	0.01	0.03
Time to the first event HR	0.28	0.14	0.29	0.14	0.30	0.15
Marginal model HR	0.03	0.10	0.04	0.10	0.04	0.10
Andersen-Gill HR	−0.07	0.09	−0.05	0.10	−0.05	0.10
Ratio of means	0.01	0.04	0.01	0.05	0.01	0.05
Ratio of medians	0.01	0.06	0.02	0.07	0.01	0.06

*SD* standard deviation

^a^Rate ratio from negative binomial regression model

^b^Mean of the estimate minus negative binomial rate ratio

### Convergence

The negative binomial regression did not always converge and in these instances yielded the same intervention estimates as the Poisson regression (Table 6). Non-convergence was greatest in simulation scenarios where there was no overdispersion and was therefore unlikely to affect the results of the simulations.

**Table 6:** Rates of non-convergence for negative binomial regression from the 10,000 simulations

	Very low mean (%)	Low mean (%)	Moderate mean (%)	High mean (%)
No overdispersion	8.15	6.99	1.37	0.04
Moderate overdispersion	3.77	5.28	0.33	0
High overdispersion	2.62	4.29	0.20	0

The ratio of medians was impossible to calculate for the very low mean, and for the low mean, the ratio of medians was only possible for 292, 717 and 2087 of the 10,000 simulations for no, moderate and high overdispersion, respectively.

### The empirical case study

Eight (of the possible 43) data sets were provided, each containing (by participant) the number of falls during the trial, group allocation (exercise, no exercise), and the time period of falls were monitored [15, 19–25]. Some details of these eight studies are presented in Table 7.

**Table 7:** Characteristics of studies in the empirical study

Study name	Follow-up	Interventions	Number in arm	Number of falls
Barnett et al. 2003 [19]	12 months	Group exercise with home exercise plan	76	40
		No exercise	74	70
Campbell et al. 1997 [20]	24 months	Individualised supervised home exercise	116	88
		No treatment	117	152
Campbell et. al 1999 [21]	10 months	Individualised supervised home exercise	45	22
		No treatment	48	35
Green et al. 2002 [22]	9 months	Individualised physiotherapy exercise in the community	85	74
		No treatment	85	51
Lord et al. 1995 [23]	12 months	Exercise classes	75	44
		No treatment	94	75
Lord et al. 2003 [19]	12 months	Group exercise	259	174
		No treatment or relaxation exercises	249	211
Robertson et al. 2001 [24]	12 months	Individualised supervised home exercise	121	80
		No exercise	119	109
Skelton et al. 2005 [25]	36 weeks	Individualised supervised group and home exercise	50	66
		Pamphlet on home exercises	31	119

**Fig. 5:**
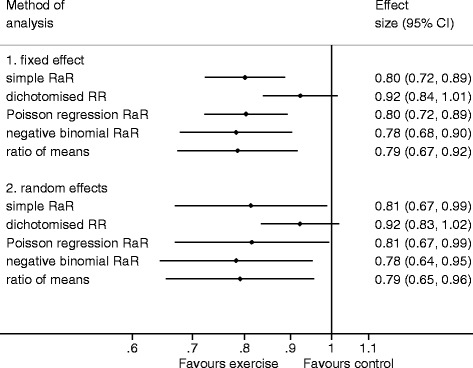
Pooled estimates of the effect of exercise in the prevention of falls using different methods of analysis calculated using individual patient data from eight studies

Meta-analysing risk ratios from dichotomised outcomes yielded estimates of intervention effect that differed from those from the other analytical methods (Fig. 5). The other methods all provided very similar results. There was little variation in the precision of the fixed effect estimate across the analytical methods. However, the 95 % CI of the random effects estimate based on the dichotomised outcomes was notably narrower than the 95 % CI for other random effects analyses.

### No difference between groups

When there was no difference in the effect of treatment in the groups, all methods gave very similar results for all scenarios.

## Discussion

The results of this study suggest that it may well be possible in many situations to combine in a meta-analysis the estimates of intervention effects for count outcomes analysed in various ways, as the results from the different analysis methods were very similar. Apart from a few instances, most analyses gave estimates that were on average close to the RaR from a negative binomial regression. Further, examination of the range of data from the simulations showed that the confidence intervals of most of the methods were similar. Therefore, pooling intervention estimates calculated by different methods is likely to be generally reasonable. This has been shown using both simulations and actual data from a meta-analysis of RCTs. When events were rare, or there was no treatment effect, all methods of analysis provide a very similar estimate of intervention effect with similar variation. An exception to this is the ratio of medians, which is impossible to calculate unless both groups have more than 50 % of participants with events. As events become more common, dichotomising the results into those with the event and those without increasingly loses the ability to discriminate between treatments, and the confidence interval becomes narrower. Intuitively, as events become more common, it is likely that all, or almost all, of the participants will experience one or more events. Similarly, time to the first event loses the ability to discriminate with increasing event rates, but this happens more slowly than with dichotomising the data.

Poisson regression and negative binomial regression models gave very similar results for the RaR, even when there was a significant amount of overdispersion. This was expected given these distributions have the same expected value [13, 26]. The standard error of the RaR estimated from Poisson regression will be too small in the presence of overdispersion, which will have implications for the weights in meta-analytic models. In this simulation, the underestimation of the standard error was only slight but was most noticeable with both a high mean and a lot of overdispersion. Trials that are analysed using Poisson regression in the presence of overdispersion will receive too much weight in the meta-analysis. The impact of not allowing for overdispersion, and subsequent underestimation of the variance of the intervention effect, was evident when comparing the fixed effect meta-analysis confidence intervals calculated from using Poisson regression compared with the negative binomial regression in the empirical study.

Adjusting the survival analyses for multiple events also gave estimates close to those from the negative binomial regression, although the confidence intervals were wider, especially as the mean increases. An exception to this was the Andersen-Gill method that gave an estimate of the HR that was, on average, slightly further from 1 than the negative binomial RaR. The difference between the estimates increases as the mean increases, which may lead to a different interpretation of the intervention effect and make it unreasonable to combine Andersen-Gill HR estimates with those estimated from the negative binomial regression. All survival models in these simulations make the assumption of proportional hazards. In our simulations, the proportional hazards assumption is likely to be true because of the way the data was generated but may not be so for any particular RCT.

The ratio of medians is clearly inappropriate where the event rate is low as the medians in one or both groups are likely to be zero. As the event rate increases, the average difference between estimates calculated from the ratio of medians and negative binomial regression is small. However, in any particular trial, the difference could be large, as indicated by the large standard deviation of the differences. Especially when the mean is low, the distribution of the ratio of medians is highly concentrated at discrete values but becomes smoother as the mean increases. This could lead to different variances compared with the other models. In practice, it is difficult to use the ratio of medians as the standard error cannot be computed from commonly reported statistics. There is a formula for the 95 % confidence interval of the ratio of medians, but calculation requires the original data [27]. An alternative to using this formula, but still requiring the original data, is to use a method such as bootstrapping to compute the standard errors. More commonly, trial authors will report one of the other effect measures, such as the simple RaR (or at least the raw data that allows this ratio to be calculated). Calculation of the ratio of means is likely to be possible from many studies where the means are reported. There is a standard formula that calculates an approximate standard error from the mean, standard deviation and number of individuals in each of the arms of the study [4].

It is perhaps unsurprising that the estimates and their distributions are similar. The simple RaR and Poisson regression estimate the same parameter; any differences are likely to be due to rounding errors, as the Poisson regression requires more calculations to be performed. The expected values of the estimates from Poisson regression and negative binomial regression are the same. Survival analysis and Poisson regression estimate the same parameter when the baseline hazard is constant [28], which in these simulations will hold, and should for many RCTs. The ratio of means is the coefficient from a linear regression of group assignment on the log of the count outcomes. This is similar to the coefficient in a Poisson regression, except that linear regression does not cope well with zero scores in the outcome, the error structure is different and it is unable to adjust for different follow-up periods.

We chose the negative binomial model as the reference model as it seems appropriate for this sort of data, especially in the presence of overdispersion. This does not allow for the estimation of bias in any of the methods, as we do not have the “true” value. As the question we wanted to answer was whether the results of the different methods could be combined in a meta-analysis looking at the difference from one of the methods was more appropriate.

There are other possibilities for the analysis of count outcomes, such as zero inflated Poisson, zero inflated negative binomial and Poisson regression with robust errors which allows for overdispersion by relaxing the requirement that the mean and variance are equal. However, we did not evaluate these methods since they are not used very often in practice.

Previously, it has been established that, to prevent bias, it is important to account for the length of exposure, which may differ because of dropouts that are not missing at random [12, 29]. The simple rate ratio, Poisson regression and negative binomial regression are all able to adjust for varying follow-up times, as do the survival analysis methods. Thus, it is surprising that the ratio of means and the ratio of medians yield similar effect estimates to those estimated from the negative binomial regression. This may be a result of the data sets generated assuming similar attrition across groups, and the missing data mechanism being participants missing completely at random. Under various scenarios (e.g. varying attrition rates and different missing data mechanisms (e.g. not missing at random)), the ratio of means and ratio of medians may yield effect estimates that differ compared with those estimated from negative binomial regression.

The choice of a uniform distribution to pick the times that the events occurred may not be the most realistic option. Events may be more likely to occur closer together or further apart than a uniform distribution would give. They also may not be independent of each other, particularly as having an event may increase or decrease the time to the next event and this may depend on the nature of the event.

The fact that intervention effect estimates from RCTs using different analytical methods can, in some circumstances, be pooled in a meta-analysis should not make the method of analysis a random choice in any particular trial. The analysis should match the hypothesis and the study design. We have previously advocated for the use of negative binomial regression in evaluating falls prevention studies [11], as have others for this type of data [14]. Negative binomial regression allows for all events to be included (thus using all information) and the length of exposure to vary and more appropriately accounts for overdispersed data. But it does treat individuals who have multiple events in quick succession, and then none for the rest of the follow-up period the same as those who have the same number of events evenly spread out throughout the period.

We have concentrated on the point estimates, with no detailed examination of the variances of these. Thus, more questions remain to be answered about meta-analysis of count data outcomes analysed using alternative methods. The impact of the trial analytical method on meta-analytic intervention effects, their standard errors and heterogeneity needs to be investigated. The impact is likely to vary by the chosen meta-analysis model (random effects versus fixed effect), so any investigation should examine both models. This simulation only examined data that were missing completely at random. This is overly simplistic, and research examining the impact of different missing data mechanisms and how these interact with the trial and meta-analysis methods would be valuable.

The focus of this paper is on RCTs, but these methods of analysis are used for other types of studies (non-randomised trials, observational studies), which may also be included in meta-analyses. For study types other than RCTs, it would be critical to examine the impact of covariates and missing data, in addition to the examination we have undertaken in this paper.

## Conclusions

We have shown in this simulation study, that analysing outcomes using different methods yielded estimates of intervention effect that were similar in both average estimates and variances. When the mean of the counts is more than 0.5, analyses using dichotomisation or time to the first event should not be pooled with intervention effects estimated from other methods. Dichotomising, when the event rate is at this level or higher, may not be an appropriate method for analysing individual studies as it is likely to underestimate treatment differences as well as giving confidence intervals that are too narrow.

## Additional file

Additional file 1:
**The implications for meta-analysis of alternative methods for analysing count data.** Additional file 1 contains three sections. The first is example Stata code for the simulations to make it absolutely clear how they were done. It is possible to present the results by using ratios of the results for the difference method rather than difference. These are presented in the second section. The third section contains the plots of the distributions of the differences for all the simulated scenarios. (DOCX 1,488 kb)

## Abbreviations

CI: confidence interval
HR: hazard ratio
RaR: rate ratio
RCT: randomised controlled trial
RR: risk ratio
