# Supplementary material for: Do alternative methods for analysing count data produce similar estimates? Implications for meta-analyses
Source: Syst Rev. 2015 Nov 17;4:163. doi: 10.1186/s13643-015-0144-x (PMC4650317; doi:10.1186/s13643-015-0144-x)
Supplement: Additional file 1: — The implications for meta-analysis of alternative methods for analysing count data. Additional file 1 contains three sections. The first is example Stata code for the simulations to make it absolutely clear how they were done. It is possible to present the results by using ratios of the results for the difference method rather than difference. These are presented in the second section. The third section contains the plots of the distributions of the differences for all the simulated scenarios. (DOCX 1,488 kb) [file 13643_2015_144_MOESM1_ESM.docx]

Online Supplement for “The implications for meta-analysis of alternative methods for analysing count data”

Section 1.

Example of Stata code for one of the simulations.

clear

set seed 2175333

set more off

cd "/Users/peterherbison/Documents/Current work/Myself/Count data and falls/Simulations/More replications"

file open results using simulate3b_multiple_day.raw, write text replace

file write results "simulation" _tab "calcrr" _tab "fromir" _tab "event" _tab "poisson" _tab

file write results "nbreg" _tab "converged" _tab "contmedian" _tab "exptmedian" _tab

file write results "contmean" _tab "exptmean" _tab "meandiff" _tab "sediff" _tab "hazardfirst" _tab

file write results "hazardmultiple1" _tab "hazardmultiple2"_n

forvalues x=1/10000 {

clear

file write results (`x') _tab

local cnobs=round(rnormal(100,2))

set obs `cnobs'

gen id=_n

gen treat="control"

gen unirand1=runiform()

gen unirand2=runiform()

gen unirand3=runiform()

gen nevents=rpoisson(7)

gen duration=365 if unirand2<0.8

replace duration=round(unirand3*365) if unirand2>=0.8

local expobs=round(rnormal(100,2))

local totobs=`cnobs' + `expobs'

set obs `totobs'

replace treat="experimental" if treat==""

replace id=_n if treat=="experimental"

replace unirand1=runiform() if treat=="experimental"

replace unirand2=runiform() if treat=="experimental"

replace unirand3=runiform() if treat=="experimental"

replace nevents=rpoisson(5) if treat=="experimental"

replace duration=365 if unirand2<0.8 & treat=="experimental"

replace duration=round(unirand3*365) if unirand2>=0.8 & treat=="experimental"

* Generate all times to events

tab nevents

scalar define maxev=r(r)

forvalues y=1/100 {

if `y'<=maxev {

gen timeevent`y'=.

gen trand`y'=runiform()

replace timeevent`y'=duration if `y'==nevents+1

replace timeevent`y'=round(trand`y'*duration) if `y'<nevents+1

replace timeevent`y'=0.1 if timeevent`y'==0

drop trand`y'

}

}

* start analyses

tabstat nevents duration, by(treat) statistics(sum) save

matrix cont=r(Stat1)

matrix expt=r(Stat2)

scalar contrate=cont[1,1]/cont[1,2]

scalar exptrate=expt[1,1]/expt[1,2]

scalar simplerr=exptrate/contrate

* simple rate ratio - simplerr

file write results (simplerr) _tab

gen ntreat=0 if treat=="control"

replace ntreat=1 if treat=="experimental"

ir nevents ntreat duration

* from ir - r(irr)

file write results (r(irr)) _tab

gen event=nevents

replace event=1 if event>0

cs event ntreat

* relative risk for those with events - r(rr)

file write results (r(rr)) _tab

gen duration1=duration

replace duration1=0.1 if duration==0

xi:poisson nevents i.treat, irr exposure(duration1)

matrix irrcoef1=e(b)

* poisson regression rate ratio - exp(irrcoef1[1,1])

file write results (exp(irrcoef1[1,1])) _tab

xi:nbreg nevents i.treat, irr iterate(150) exposure(duration1)

matrix irrcoef2=e(b)

* neg binomial regression - exp(irrcoef2[1,1])

file write results (exp(irrcoef2[1,1])) _tab (e(converged)) _tab

gen evpertime=nevents/365 if duration==365

replace evpertime=nevents/(duration+1) if duration<365

tabstat evpertime, by(treat) statistics(median) save

matrix contmedian=r(Stat1)

matrix exptmedian=r(Stat2)

file write results (contmedian[1,1]) _tab (exptmedian[1,1]) _tab

ttest nevents, by(treat)

file write results (r(mu_1)) _tab (r(mu_2)) _tab (r(mu_1) - r(mu_2)) _tab (r(se)) _tab

stset timeevent1, failure(event==1)

xi:stcox i.treat

matrix hrcoef=e(b)

* HR to first event

file write results (exp(hrcoef[1,1])) _tab

stset, clear

drop unirand1 unirand2 unirand3 _Itreat_2

reshape long timeevent, i(id) j(eventnum)

drop if timeevent==.

sort id timeevent

replace event=0 if id!=id[_n+1]

stset timeevent, failure(event==1) id(id)

xi:stcox i.treat

matrix hrcoef=e(b)

* HR from marginal model

file write results (exp(hrcoef[1,1])) _tab

stset, clear

gen timebetween=timeevent if id!=id[_n-1]

replace timebetween=timeevent-timeevent[_n-1] if id==id[_n-1]

stset timebetween, failure(event==1) id(id)

xi:stcox i.treat

matrix hrcoef1=e(b)

* HR from Andersen-Gill

file write results (exp(hrcoef1[1,1])) _n

}

file close results

Section 2.

An alternative to looking at the differences in estimates between the reference method (negative binomial regression) and the other methods is to examine the ratios of the two effects. A ratio of 1.03 is interpreted as a 3% increase.

**Online Table 1.** Simulation results, expressed as ratios of rates, for the very low mean (control 0.2, treatment 0.15)

|  | No overdispersion  RaR* = 0.79 | | Moderate overdispersion  RaR* = 0.73 | | High overdispersion  RaR* = 0.72 | |
| --- | --- | --- | --- | --- | --- | --- |
|  | Ratio† | SD‡ | Ratio | SD | Ratio | SD |
| Dichotomise RR | 1.03 | 0.11 | 1.09 | 0.13 | 1.08 | 0.13 |
| Poisson RaR | 1.00 | 0.01 | 1.00 | 0.02 | 1.00 | 0.02 |
| Simple RaR | 1.00 | 0.01 | 1.00 | 0.02 | 1.00 | 0.02 |
| Time to first event HR | 1.01 | 0.11 | 1.03 | 0.13 | 1.04 | 0.13 |
| Marginal model HR | 1.01 | 0.11 | 1.03 | 0.13 | 1.04 | 0.12 |
| Andersen-Gill HR | 1.01 | 0.11 | 1.03 | 0.13 | 1.03 | 0.12 |
| Ratio of means | 1.00 | 0.04 | 1.03 | 0.04 | 1.01 | 0.05 |
| Ratio of medians | Not possible as both medians are zero | | | | | |

* Rate ratio from negative binomial regression model

† Ratio of the estimate to the negative binomial rate ratio

‡ SD – Standard Deviation

**Online Table 2.** Simulation results, expressed as ratios of rates, for the low mean (control 0.5, treatment 0.35)

|  | No overdispersion  RaR* = 0.71 | | Moderate overdispersion  RaR* = 0.73 | | High overdispersion  RaR* = 0.74 | |
| --- | --- | --- | --- | --- | --- | --- |
|  | Ratio† | SD‡ | Ratio | SD | Ratio | SD |
| Dichotomise RR | 1.08 | 0.13 | 1.09 | 0.12 | 1.09 | 0.12 |
| Poisson RaR | 1.00 | 0.02 | 1.00 | 0.01 | 1.00 | 0.01 |
| Simple RaR | 1.00 | 0.02 | 1.00 | 0.01 | 1.00 | 0.01 |
| Time to first event HR | 1.04 | 0.13 | 1.02 | 0.12 | 1.02 | 0.12 |
| Marginal model HR | 1.04 | 0.12 | 1.02 | 0.11 | 1.01 | 0.11 |
| Andersen-Gill HR | 1.03 | 0.12 | 1.01 | 0.11 | 1.01 | 0.11 |
| Ratio of means | 1.01 | 0.05 | 1.00 | 0.04 | 1.00 | 0.04 |
| Ratio of medians | Only possible for 292, 717 and 2087 of the 10000 simulations respectively | | | | | |

* Rate ratio from negative binomial regression model

† Ratio of the estimate to the negative binomial rate ratio

‡ SD – Standard Deviation

**Online Table 3.** Simulation results, expressed as ratios of rates, for the moderate mean (control 2, treatment 1.5)

|  | No over dispersion  RaR* = 0.75 | | Moderate overdispersion  RaR* = 0.77 | | High overdispersion  RaR* = 0.79 | |
| --- | --- | --- | --- | --- | --- | --- |
|  | Ratio† | SD‡ | Ratio | SD | Ratio | SD |
| Dichotomise RR | 1.21 | 0.12 | 1.19 | 0.12 | 1.19 | 0.12 |
| Poisson RaR | 1.00 | 0.01 | 1.01 | 0.01 | 1.01 | 0.02 |
| Simple RaR | 1.00 | 0.01 | 1.01 | 0.01 | 1.01 | 0.02 |
| Time to first event HR | 1.09 | 0.14 | 1.09 | 0.15 | 1.09 | 0.15 |
| Marginal model HR | 1.02 | 0.12 | 1.02 | 0.12 | 1.02 | 0.12 |
| Andersen-Gill HR | 0.98 | 0.11 | 0.98 | 0.11 | 0.98 | 0.11 |
| Ratio of means | 1.00 | 0.04 | 1.01 | 0.05 | 1.01 | 0.05 |
| Ratio of medians | 0.92 | 0.25 | 1.11 | 0.23 | 1.08 | 0.19 |

* Rate ratio from negative binomial regression model

† Ratio of the estimate to the negative binomial rate ratio

‡ SD – Standard Deviation

**Online Table 4.** Simulation results, expressed as ratios of rates, for the high mean (control 7, treatment 5)

|  | No overdispersion  RaR* = 0.71 | | Moderate overdispersion  RaR* = 0.70 | | High overdispersion  RaR* = 0.70 | |
| --- | --- | --- | --- | --- | --- | --- |
|  | Ratio† | SD‡ | Ratio | SD | Ratio | SD |
| Dichotomise RR | 1.42 | 0.12 | 1.44 | 0.14 | 1.44 | 0.14 |
| Poisson RaR | 1.02 | 0.03 | 1.02 | 0.04 | 1.02 | 0.05 |
| Simple RaR | 1.02 | 0.03 | 1.02 | 0.04 | 1.02 | 0.05 |
| Time to first event HR | 1.40 | 0.21 | 1.42 | 0.22 | 1.44 | 0.23 |
| Marginal model HR | 1.05 | 0.14 | 1.06 | 0.15 | 1.07 | 0.15 |
| Andersen-Gill HR | 0.91 | 0.13 | 0.93 | 0.14 | 0.93 | 0.15 |
| Ratio of means | 1.02 | 0.06 | 1.02 | 0.07 | 1.02 | 0.08 |
| Ratio of medians | 1.01 | 0.09 | 1.03 | 0.10 | 1.02 | 0.09 |

* Rate ratio from negative binomial regression model

† Ratio of the estimate to the negative binomial rate ratio

‡ SD – Standard Deviation

If the distributions of the results from the different methods are very unlike each other then it may be difficult to decide whether they could possibly be conbined. The following figures are histograms of the 10,000 results from each of the combinations of mean and overdispersion.

Online figure 1a. Histogram of simulation results for the very low mean (control 0.2, treatment 0.15) with no overdispersion

Online figure 1b. Histogram of simulation results for the very low mean (control 0.2, treatment 0.15) with moderate overdispersion

Online figure 1c. Histogram of simulation results for the very low mean (control 0.2, treatment 0.15) with high overdispersion

Online figure 2a. Histogram of simulation results for the low mean (control 0.5, treatment 0.35) with no overdispersion

Online figure 2b. Histogram of simulation results for the low mean (control 0.5, treatment 0.35) with moderate overdispersion

Online figure 2c. Histogram of simulation results for the low mean (control 0.5, treatment 0.35) with high overdispersion

Online figure 3a. Histogram of simulation results for the moderate mean (control 2, treatment 1.5) with no overdispersion

Online figure 3b. Histogram of simulation results for the moderate mean (control 2, treatment 1.5) with moderate overdispersion

Online figure 3c. Histogram of simulation results for the moderate mean (control 2, treatment 1.5) with high overdispersion

Online figure 4a. Histogram of simulation results for the high mean (control 7, treatment 5) with no overdispersion

Online figure 4b. Histogram of simulation results for the high mean (control 7, treatment 5) with moderate overdispersion

Online figure 4c. Histogram of simulation results for the high mean (control 7, treatment 5) with high overdispersion
